# Chemogenetic Activation of Feed-Forward Inhibitory Parvalbumin-Expressing Interneurons in the Cortico-Thalamocortical Network During Absence Seizures

**DOI:** 10.3389/fncel.2021.688905

**Published:** 2021-05-28

**Authors:** Sandesh Panthi, Beulah Leitch

**Affiliations:** Department of Anatomy, School of Biomedical Sciences, Brain Health Research Centre, University of Otago, Dunedin, New Zealand

**Keywords:** cortico-thalamocortical, parvalbumin, GABAergic interneurons, feed-forward inhibition, DREADDs, absence seizures, pentylenetetrazol

## Abstract

Parvalbumin-expressing (PV+) interneurons are a subset of GABAergic inhibitory interneurons that mediate feed-forward inhibition (FFI) within the cortico-thalamocortical (CTC) network of the brain. The CTC network is a reciprocal loop with connections between cortex and thalamus. FFI PV+ interneurons control the firing of principal excitatory neurons within the CTC network and prevent runaway excitation. Studies have shown that generalized spike-wave discharges (SWDs), the hallmark of absence seizures on electroencephalogram (EEG), originate within the CTC network. In the stargazer mouse model of absence epilepsy, reduced FFI is believed to contribute to absence seizure genesis as there is a specific loss of excitatory α-amino-3-hydroxy-5-methyl-4-isoxazolepropionic acid receptors (AMPARs) at synaptic inputs to PV+ interneurons within the CTC network. However, the degree to which this deficit is directly related to seizure generation has not yet been established. Using chemogenetics and *in vivo* EEG recording, we recently demonstrated that functional silencing of PV+ interneurons in either the somatosensory cortex (SScortex) or the reticular thalamic nucleus (RTN) is sufficient to generate absence-SWDs. Here, we used the same approach to assess whether activating PV+ FFI interneurons within the CTC network during absence seizures would prevent or reduce seizures. To target these interneurons, mice expressing Cre recombinase in PV+ interneurons (PV-Cre) were bred with mice expressing excitatory Gq-DREADD (hM3Dq-flox) receptors. An intraperitoneal dose of pro-epileptic chemical pentylenetetrazol (PTZ) was used to induce absence seizure. The impact of activation of FFI PV+ interneurons during seizures was tested by focal injection of the “designer drug” clozapine N-oxide (CNO) into either the SScortex or the RTN thalamus. Seizures were assessed in PV^Cre^/Gq-DREADD animals using EEG/video recordings. Overall, DREADD-mediated activation of PV+ interneurons provided anti-epileptic effects against PTZ-induced seizures. CNO activation of FFI either prevented PTZ-induced absence seizures or suppressed their severity. Furthermore, PTZ-induced tonic-clonic seizures were also reduced in severity by activation of FFI PV+ interneurons. In contrast, administration of CNO to non-DREADD wild-type control animals did not afford any protection against PTZ-induced seizures. These data demonstrate that FFI PV+ interneurons within CTC microcircuits could be a potential therapeutic target for anti-absence seizure treatment in some patients.

## Introduction

Childhood absence epilepsy (CAE) is the most common form of pediatric epilepsy, which is characterized by synchronous 3–4 Hz generalized spike-wave discharges (SWDs) associated with impaired awareness. Absence seizures are known to arise from altered dynamics within the cortico-thalamocortical (CTC) network (McCormick and Contreras, 2001; Crunelli and Leresche, 2002; Maheshwari and Noebels, 2014; Lüttjohann and van Luijtelaar, 2015; Crunelli et al., 2020) but the precise cellular and molecular mechanisms are not fully understood and appear to be multifactorial. Within the CTC network, feed-forward inhibition (FFI) is essential to prevent runaway excitation and is mediated by fast-spiking parvalbumin-expressing (PV+) inhibitory interneurons. Studies conducted using the well-established stargazer mouse model of absence epilepsy have shown defects in α-amino-3-hydroxy-5-methyl-4-isoxazolepropionic acid receptor (AMPAR) expression at excitatory synapses in feed-forward inhibitory PV+ interneurons in the somatosensory cortex (SScortex) (Maheshwari et al., 2013; Adotevi and Leitch, 2016, 2017, 2019) and reticular thalamic nuclei (RTN) (Menuz and Nicoll, 2008; Barad et al., 2012) of the CTC network. The loss of synaptic AMPARs is the result of a genetic mutation in stargazin, a transmembrane AMPAR regulatory protein (TARP) (Noebels et al., 1990; Letts et al., 1998) that traffics AMPARs to the synapse. However, the extent to which this defect weakens FFI within the CTC network and whether this directly contributes toward the generation and maintenance of absence seizures had not been previously established. We recently reported that acute selective silencing of PV+ interneurons in the CTC network (either in the SScortex or the RTN thalamus) impairs FFI and generates absence-like SWDs in normal non-epileptic mice (Panthi and Leitch, 2019). In the current study, the goal was to determine the impact of selectively activating PV+ inhibitory interneurons within the CTC network during absence seizures, to determine if this was sufficient to prevent or reduce seizure activity.

To investigate the consequences of activating feed-forward inhibitory PV+ interneurons *in vivo*, we used Designer Receptors Exclusively Activated by Designer Drugs (DREADD) based technology (Armbruster et al., 2007). DREADDs are mutationally modified muscarinic acetylcholine (ACh) receptors, which can be specifically expressed in a targeted cell population and exclusively activated by the designer drug clozapine N-oxide (CNO), but not by their endogenous ligand ACh (Armbruster et al., 2007; Rogan and Roth, 2011). In this study, we used excitatory Gq-DREADDs inserted into PV+ interneurons to selectively activate FFI (Alexander et al., 2009). Cre-dependent excitatory Gq-DREADD mice (i.e., hM3Dq-flox mice) were crossed with PV-Cre mice to express Gq-DREADDs in PV+ interneurons (Zhu et al., 2016). Selective activation of FFI within the CTC network was achieved by focal injection of CNO into cortical or thalamic regions of interest (Panthi and Leitch, 2019).

To induce absence seizure in PV^Cre^/Gq-DREADD mice we used pentylenetetrazol (PTZ). This chemical is routinely used in epileptic studies to induce both absence and generalized tonic-clonic seizures (Snead, 1992; Velíšková et al., 2017) and has been used in the screening of antiepileptic drugs since 1970s (see reviews by Krall et al., 1978 and Löscher, 2011). PTZ impairs GABA mediated inhibition by antagonizing GABA_A_ receptors (Huang et al., 2001). The severity of PTZ induced seizures is dose dependent. Low dose PTZ administration (i.e., ∼20 mg/kg) is an established experimental method to pharmacologically induce generalized absence seizures involving thalamocortical mechanisms whereas high doses (>40 mg/kg) induce spike trains with tonic-clonic seizures (Snead, 1992; Snead et al., 2000; Cortez et al., 2016).

To date, there have been relatively few studies investigating the impact of activating PV+ interneurons during PTZ-induced seizures. Clemente-Perez et al. (2017) used PTZ treatment combined with optogenetic activation of PV+ interneurons to selectively modulate PV+ neurons in the RTN thalamus during free behavior. They found that unilateral optical stimulation of RTN PV+ interneurons during PTZ-induced seizures disrupts bilateral generalized seizures in PV-Cre mice injected with channelrhodopsin-2 (ChR2). Another recent DREADD-based study indicated that global activation of PV+ interneurons increases the latency and decreases the susceptibility of PTZ-induced tonic-clonic and myoclonic seizures (Johnson et al., 2018). Other studies have used different chemicals to study the impact of activating PV+ interneurons during pharmacologically induced seizures. For example, Assaf and Schiller (2016) demonstrated that optogenetic activation of cortical PV+ interneurons causes the termination of 4-aminopyridine (4-AP) induced spontaneous electrographic seizures. Activating hippocampal PV+ interneurons, on the other hand, attenuates temporal lobe seizures induced by kainic acid (KA) (Krook-Magnuson et al., 2013; Wang et al., 2018). Additionally, parvalbumin knockout (PV^–/–^) mice have higher susceptibility to chemically induced seizures and these animals experience more severe seizures compared to wild-type (PV^+/+^) controls (Schwaller et al., 2004). However, there have been no published studies to date reporting the impact of activating PV+ interneurons within the CTC network during absence seizures using DREADD technology.

Hence, the aim of the current study was to test the impact of activating feed-forward inhibitory PV+ interneurons within the CTC network of PV^Cre^/Gq-DREADD mice during PTZ-induced absence seizures, using simultaneous video/electroencephalogram (EEG) recording. We hypothesized that activating PV+ interneurons within the SScortex or the RTN thalamus would prevent or reduce PTZ-induced absence seizures.

## Materials and Methods

### Animals and Breeding Paradigm

Experiments were performed on adult male and female double-transgenic mice expressing excitatory Gq-DREADD receptors in PV+ interneurons. Cre-recombinase conditional excitatory Gq-DREADD (i.e., hM3Dq-flox mice) and PV-Cre knockin mice were obtained from Jackson Laboratories, United States. Both transgenic mice were created on the background of C57BL/6 strain (Zhu et al., 2016). Detailed descriptions of hM3Dq-flox and PV-Cre knockin mouse lines can be found in Jackson laboratories datasheets stock no. 026220^1^ and stock no. 008069^2^, respectively. Homozygous female PV-Cre mice were crossed with heterozygous hM3Dq-floxed males to generate litters with PV^Cre^/Gq-DREADD and non-DREADD expressing wild-type (WT) control littermates, as illustrated in Supplementary Figure 1A. The hM3Dq-floxed mice have a *loxP*-flanked STOP cassette designed to prevent transcription of the downstream HA-hM3Dq-pta-mCitrine coding region (Supplementary Figure 1A). Mating these strains (PV-Cre and hM3Dq-floxed) removes the *loxP*-flanked STOP cassette only in the cell type specified by the Cre-recombinase system (Zhu et al., 2016). This allows the strong expression of hemagglutinin (HA)-tag only in PV+ interneurons. In this study, only female PV-Cre mice were crossed with male hM3Dq-flox mice to avoid unwanted germline recombination. Mice were bred and housed at the University of Otago Animal Facility at a controlled room temperature (22–24°C) with *ad libitum* access to food and water.

### Genotyping

Genotyping was performed to verify the mouse genotype. Ear notches were collected from offspring of PV-Cre × hM3Dq-flox mice. They were mixed in DNA lysis buffer and proteinase K (Roche, Basel) and digested overnight at 55°C. The following day samples were centrifuged and DNA was obtained to process for PCR. Genotyping was performed separately to confirm Cre knockin and hM3Dq-flox using the following Cre, hM3Dq mutant and wild-type primers (Integrated DNA technologies, United States): CCT GGA AAA TGC TTC TGT CCG Cre-forward; CAG GGT GTT ATA AGC AAT CCC reverse for Cre allele; CGC CAC CAT GTA CCC ATA C hM3Dq-flox forward; GTG GTA CCG TCT GGA GAG GA reverse for hM3Dq-flox allele; AAG GGA GCT GCA GTG GAG TA wild-type forward; CCG AAA ATC TGT GGG AAG TC reverse for wild-type allele. PCR product was allowed to run in agarose gel at 70–80 V for around 2 h. The gel was then placed in a UV light source to view and photograph bands. Supplementary Figure 1B is a representative image of gel electrophoresis blot for the PV-Cre knockin and hM3Dq-flox for three separate mice. Bands at 300 and 350 bp confirmed the PV-Cre knockin whereas bands at 204 and 300 bp indicated heterozygous hM3Dq-flox, and a band around 300 bp confirmed wild-type mice (Supplementary Figure 1B).

### Immunofluorescence Confocal Microscopy

Adult mice from PV-Cre × hM3Dq-flox colony were deeply anesthetized with an intraperitoneal (i.p.) injection of 60 mg/kg of sodium pentobarbital. Transcardial perfusion was performed with 5% heparin in 0.1 M phosphate buffered saline (PBS) followed by 4% paraformaldehyde (PFA) in 0.1 M Sorensen’s phosphate buffer. Brains were extracted and post-fixed in 4% PFA overnight at 4°C. After post-fixation, brains were washed three times in 0.1 M PBS. This was followed by cryoprotection of the brains in increasing concentration of sucrose in PBS i.e., 10% for 30 min, 20% for 30 min, and 30% at 4°C until fully infiltrated with sucrose. The cerebellum was dissected from the rest of the brain, which was then sectioned into 30 μm coronal sections. The cerebellum was also sectioned into 30 μm sagittal sections. Sectioning was performed on a freezing cryostat (Leica CM1950, Wetzlar, Germany). Sections were collected into 12-well plates containing PBS.

For immunolabelling, sections were first submerged in blocking buffer [4% Normal Goat Serum (NGS), 0.1% Bovine Serum Albumin (BSA), 0.1% Triton X-100 in PBS] for 2 h at room temperature. All sections were then incubated in a mixture of primary antibodies for 48 h at 4°C. Primary and secondary antibodies used for immunofluorescence confocal microscopy in this study are listed in Table 1. Primary antibody solution was prepared in PBS with 0.1% BSA and 0.3% Triton X-100. After incubation in primary antibodies, tissue sections were washed in PBS for 45 min (15 min × 3 times). Sections were then labeled with secondary antibodies for 12 h at 4°C. After labeling the tissues with secondary antibodies, they were washed in PBS for 30 min (10 min × 3 times). Sections were then mounted on polysine-coated glass slides and cover-slipped with mounting medium (1,4 diazabicyclo (2.2.2) octane DABCO-glycerol). Slides were left to air-dry in the dark at room temperature.

**TABLE 1:** Primary and secondary antibodies used in this study for immunofluorescence confocal microscopy.

Product	Antibody/Type	Source/Catalog No.	Dilution
Parvalbumin	Primary/Mouse monoclonal	Swant/235	1:2000
HA-tag	Primary/Rabbit polyclonal	Cell Signaling/3724S	1:500
Goat anti-rabbit	Secondary/Alexa Fluor 488	Life Technologies/11008	1:1000
Goat anti-mouse	Secondary/Alexa Fluor 568	Life Technologies/11031	1:1000

### Image Acquisition and Analysis

Images were acquired using Nikon A1+ inverted confocal laser scanning microscope. Channel configurations were set for HA-tag (magenta channel, 488 nm laser excitation) and PV (cyan channel, 568 nm laser excitation). During confocal imaging, the detector offset for each channel was kept at zero; the detector gain and laser power were optimized accordingly. Scan speed and image pixel size were also set accordingly. Images were taken of the region of interest (ROI) in the SScortex, RTN thalamus and cerebellum. All immunolabelled cells in the SScortex and cerebellum sections were counted at 10× confocal images whereas those in RTN thalamus were counted using 40× confocal images. Cell counting and analysis were performed using ImageJ (Fiji) software (version 1.51, NIH, United States).

### Surgical Implantation of Prefabricated Head Mounts and Microcannulas for EEG Recordings

Twelve-week old PV^Cre^/Gq-DREADD mice were single-housed and were handled once daily for 2 days before performing surgical manipulation. Surgery was performed after subcutaneous injection of 5 mg/kg of Carprofen (for pain control) and 2 mg/kg of Marcaine (for local anesthesia). Animals were fully anesthetized with a continuous flow of isoflurane during surgical procedures. Animals were provided with supplementary heat during surgery by placing them on a heat pad. The head of the animal was fixed with a stereotaxic frame (David Kopf Instruments, Tujunga, CA, United States). After shaving the scalp to expose the skin, a sagittal incision was made. Two pairs of holes were carefully drilled in the skull, each pair located 1.5 mm on either side of the longitudinal fissure. The first pair was located 3.5 mm anterior to bregma and the second pair 1 mm anterior to bregma. Four stainless steel screws with lead wires attached (Pinnacle Technologies, Austin, TX, United States) were inserted through these burr holes. The screw wires were then soldered to their respective channels on a prefabricated head mount (Pinnacle Technologies, Austin, TX, United States). Dental acrylic cement (Vertex Dental, Netherlands) was used to secure soldered regions.

In this study, a guide cannula (26 gauge) was implanted for focal CNO injections, based on the stereotaxic coordinates either for the SScortex (AP: −1.22 to −2.06 mm, ML: 2.8 mm) or the RTN thalamus (AP: −1.34 to −1.94 mm, ML: 2.1–2.3 mm) (Mouse Brain Atlas, Paxinos and Franklin, 3rd Edition). A dummy cannula was inserted inside the guide cannula to prevent blood or any other fluid clogging it. During CNO injections, the dummy cannula was replaced with an internal cannula (33 gauge). CNO was delivered via a Hamilton microinjection syringe attached to polythene tubing (Microtube Extrusions, Australia) and internal cannula. Guide (C315GS-2/SPC), dummy (C315IDCS-2/SPC), and internal (C315IS-2/SPC) cannulae were obtained from Plastics One Inc., Roanoke, VA, United States. Cannulae were made of stainless steel with short pedestals.

### EEG Recording

After the full recovery of animals from surgical manipulation (at least 7 days after surgery), EEG recordings were made from the subdural space over the cerebral cortex using the Pinnacle mouse system (Pinnacle Technologies, Austin, TX, United States) with simultaneous video recording. The head-mount was attached to a pre-amplifier to amplify and filter the EEG waveforms. EEG signals were filtered at 0.5 Hz high pass and 50 Hz low pass. Before each recording, animals were acclimatized in the testing environment and equipment for 1 h.

Before performing the main experiments, a pilot study was conducted to determine the dose of PTZ necessary to induce absence seizures. Briefly, a cohort of animals (*n* = 6) was injected intraperitoneally with three different doses of PTZ (10, 20, and 30 mg/kg). Based on simultaneous video/EEG data, a dose of 20 mg/kg was selected as the lowest dose that produced absence seizures in all pilot animals tested.

For experimental tests, PV^Cre^/Gq-DREADD (*n* = 14) and non-DREADD WT control (*n* = 10) animals were allocated to two different treatment groups: either SScortex cannula-implanted group (DREADD animals *n* = 7; WT controls *n* = 5) or RTN thalamus implanted group (DREADD animals *n* = 7; WT controls *n* = 5). Experiments were performed on two consecutive days. On day 1, seizures were induced in all animals (PV^Cre^/Gq-DREADD mice and non-DREADD WT control mice) by injection (i.p.) of 20 mg/kg PTZ. After 24 h, on day 2, the same cohort of animals was treated with the same dose of PTZ (i.p.) and 5 mg/kg CNO (into either the SScortex or the RTN thalamus). The timing and dose of CNO was based on our previous work (Panthi and Leitch, 2019) where 5 mg/kg CNO was lowest effective dose that activated inhibitory Gi-DREADD receptors and generated absence-like seizures in PV^Cre^/Gi-DREADD animals.

### Preparation and Delivery of CNO and PTZ

Clozapine N-oxide and PTZ were freshly prepared before every scheduled experiment. 1.5 mg of CNO (Advanced Molecular Technologies, Australia) was dissolved in 75 μl of dimethyl sulfoxide (DMSO). The volume was then adjusted to 3 ml by addition of 0.9% sterile saline to prepare CNO of 0.5 mg/ml concentration. PTZ doses of 3, 2, and 1 mg/ml concentration were prepared in 0.9% sterile saline for 30, 20, and 10 mg/kg dosage groups, respectively. PTZ was injected intraperitoneally based on the calculated dose for the body weight of the animal. On day 2, CNO (5 mg/kg) was injected focally (either into SScortex or RTN thalamus). After 10 min of baseline EEG recording, 0.3 μl of CNO was infused into the regional areas at a rate of 0.1 μl/min via Hamilton microinjection syringe. On conclusion of the experiments, mice were anesthetized and methylene blue dye was injected at the same volume and rate as CNO into the focal region of the brain under investigation to verify the CNO diffusion and localization of cannula tip and histology was performed (Supplementary Figure 2).

### Analysis of EEG Traces

Sirenia^®^ software was used for acquisition of video/EEG traces (Panthi and Leitch, 2019). Traces were manually analyzed by an investigator blind to the experimental conditions using Seizure Pro^®^ software. Bursts of oscillations were counted as absence-like SWDs if they had a spike-wave structure (spike, positive transient, and slow wave pattern), frequency of 3–8 Hz, an amplitude at least two times higher than baseline and lasted >1 s. Video was analyzed for concomitant behavioral arrest or motionless staring. EEG waveforms due to muscle activity, walking or scratching and grooming were considered as artifacts after confirmation of the behavior via simultaneous video analysis. Tonic-clonic seizures were characterized by high amplitude polyspikes lasting >1 s. Behavioral expressions for tonic-clonic seizures ranged from clonic jerking with or without loss of balance to wild jumping in some cases (Lüttjohann et al., 2009; Van Erum et al., 2019). Other types of epileptic events (such as brief generalized myoclonic jerks) different from above mentioned criteria for SWDs and tonic-clonic seizures were categorized as other types of seizures.

### Data Analysis

Statistical analyses of significant differences in the onset of seizures between PV^Cre^/Gq-DREADD and non-DREADD WT control animals were calculated using Mantel-Cox log-rank test. Comparison within the same treatment group was performed using Wilcoxon matched-pairs signed-rank test. Comparison between treatment groups was performed using Mann Whitney unpaired rank test. Data were presented as mean ± standard error of the mean (SEM). All statistical analysis was performed in GraphPad Prism 8.0 with statistical significance set at *p* < 0.05 (asterisks for *p* value: ^∗^*p* < 0.05, ^∗∗^*p* < 0.01, ^∗∗∗^*p* < 0.001, and ^****^p < 0.0001).

## Results

### Excitatory Gq-DREADD Receptors Are Expressed in Feed-Forward Inhibitory PV+ Interneurons

**FIGURE 1:**
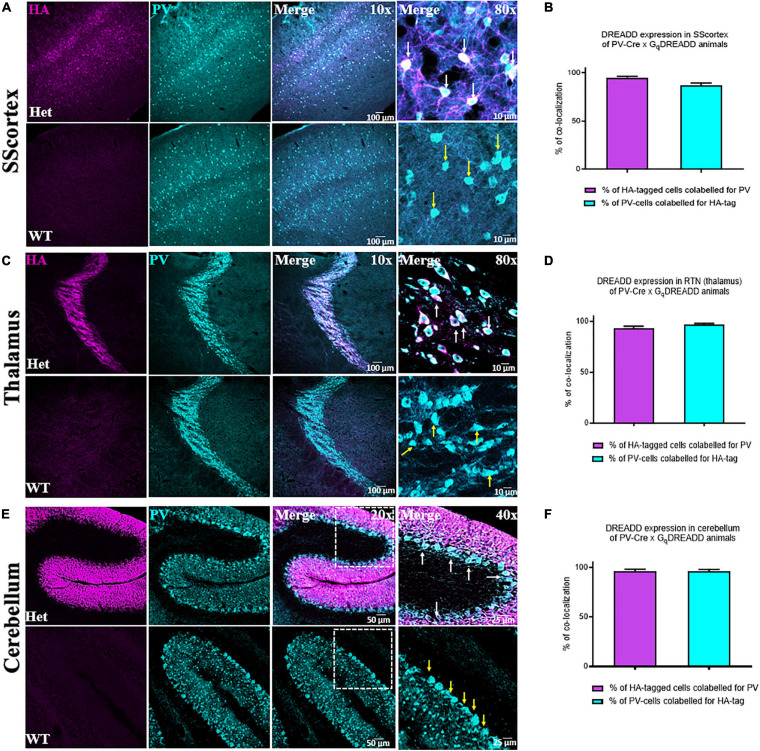
**(A,C,E)** Confocal images showing the expression of HA-tag in PV+ interneurons in the SScortex, the RTN thalamus and the cerebellum of PV^Cre^/Gq-DREADD animals, respectively. White arrows in merged images represent co-localized cells. Yellow arrows indicate PV positive neurons, which are immunonegative for HA. **(B,D,F)** Percentage of co-localization of HA-tag and PV in neurons in the SScortex, the RTN thalamus, and the cerebellum, respectively. Immunolabelled cells in the SScortex and the cerebellum were counted at 10× magnified confocal images whereas in the RTN thalamus cells were counted using 40× images (Het = PV^Cre^/Gq-DREADD offspring from homozygous PV-Cre female and heterozygous hM3Dq-flox male; WT = Non-DREADD wild type control animals).

To confirm the expression of excitatory Gq-DREADD receptors in PV+ interneurons of PV^Cre^/Gq-DREADD mice, we first performed double-labeled immunohistochemistry with antibodies against HA-tag and PV (Figure 1). HA-tag (identified by pseudo color magenta Figure 1, panel 1) was only expressed in the brain sections from PV^Cre^/Gq-DREADD mice but not non-DREADD WT control animals. PV+ interneurons (identified by pseudo color cyan Figure 1, panel 2) were highly expressed in SScortical layers II-VI, in the RTN thalamus and in the cerebellar cortex of all genotypes (Figure 1). Co-localization of HA-tag and PV (Figure 1. white arrows) was above 90% in the SScortex (Figures 1A,B), the RTN thalamus (Figures 1C,D) and the cerebellar cortex (Figures 1E,F). In the cerebellum, HA-tag was highly expressed in PV+ Purkinje soma (Figure 1E white arrows). The percentage of co-localization of HA-tag in PV+ inhibitory Purkinje cell soma was also above 90% (Figure 1F). The pattern of staining and levels of co-localization between HA-tag and PV in all three brain regions of PV^Cre^/Gq-DREADD animals were very similar to the results obtained in PV^Cre^/Gi-DREADD animals in our previous published report (Panthi and Leitch, 2019).

### 20 mg/kg IP PTZ Is Required to Induce Absence-SWDs

**FIGURE 2:**
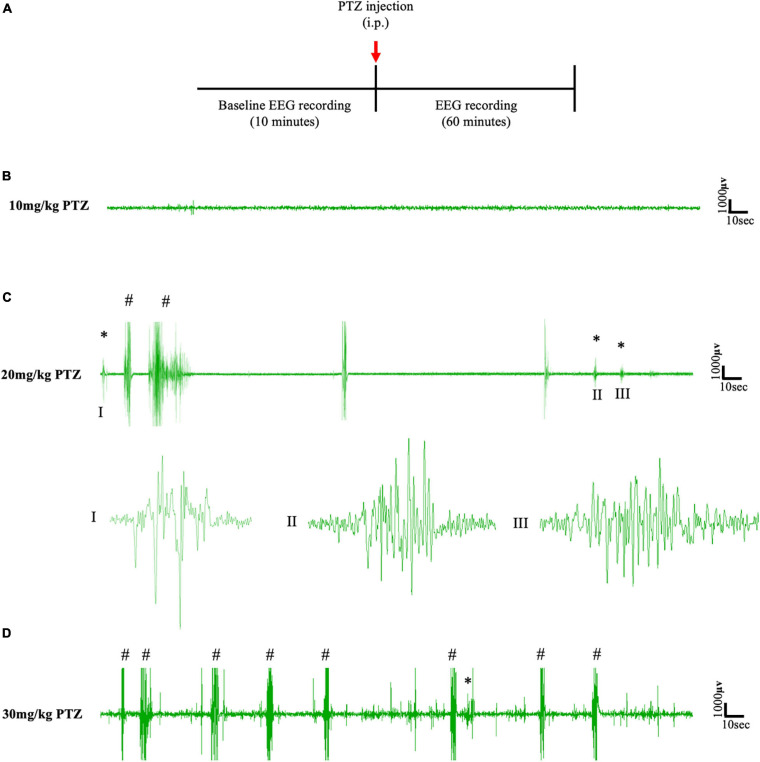
**(A)** Schematic protocol for EEG recordings before and after IP PTZ injection in the pilot study. Representative EEG traces from animals after **(B)** 10 mg/kg **(C)** 20 mg/kg **(D)** 30 mg/kg of PTZ injection. Asterisks (^∗^) and hash signs (#) represent absence-like seizures and tonic-clonic seizures, respectively. Each trace represents 5 min of EEG recording. All representative EEG traces were obtained from different animals.

To establish the dose of PTZ required to induce absence seizures, an EEG pilot study was first conducted on a cohort of animals (*n* = 6) that had not been surgically implanted with cannulae for focal delivery of CNO (Figure 2A). According to the literature, absence-SWDs can be induced in different mice and rat models using i.p. injections of PTZ at doses between 10 and 40 mg/kg (Marescaux et al., 1984; Snead et al., 2000; Girard et al., 2019; Van Erum et al., 2019). In this study, simultaneous video/EEG data showed that 10 mg/kg of PTZ did not induce seizures in any of the mice tested (Figure 2B), whereas doses of ≥30 mg/kg PTZ induced severe tonic-clonic seizures (Figure 2D). In contrast, a dose of 20 mg/kg consistently produced absence-SWDs (Figure 2C asterisks); although these were mixed with some tonic-clonic seizures (Figure 2C). As 20 mg/kg PTZ was the lowest dose that produced primarily absence seizures, it was selected as the dose injected into mice surgically implanted with either a cortical or thalamic cannula for subsequent experiments to test the impact of focally activating FFI within the CTC network during PTZ-induced absence seizures.

### Activating Feed-Forward Inhibitory PV+ Interneurons via Focal CNO Injection Suppressed PTZ-Induced Absence Seizures

**FIGURE 3:**
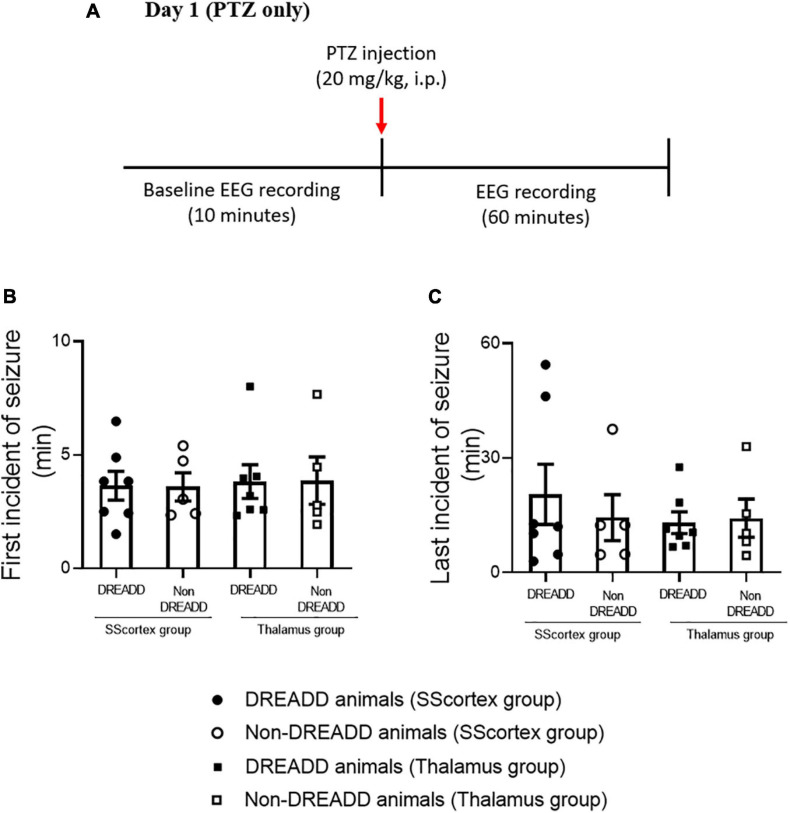
**(A)** Schematic of protocol for EEG recordings before and after PTZ injection on day 1 in experimental animals. Comparison of **(B)** onset of seizure and **(C)** last incident of seizure during 1 h of EEG recording in PV^Cre^/Gq-DREADD (DREADD) (*n* = 7) and non-DREADD (*n* = 5) WT control animals of the SScortex group and the RTN thalamus group after PTZ treatment on day 1. All values in graphs represent mean ± SEM. Comparison between treatment groups was performed using Mann Whitney unpaired rank test.

**FIGURE 4:**
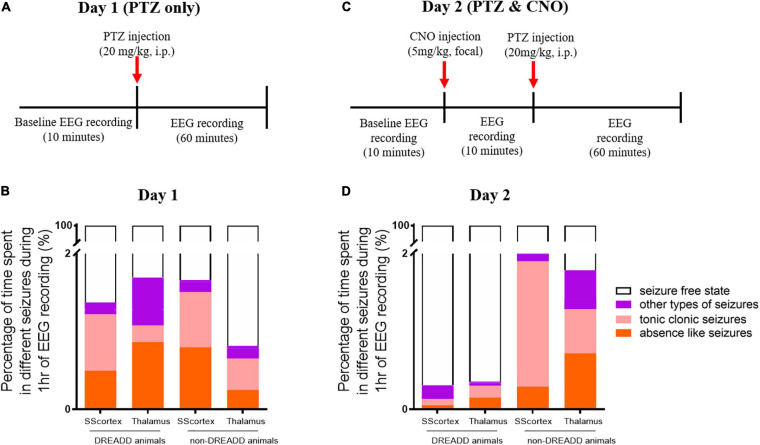
Schematic of protocol for EEG recordings before and after **(A)** PTZ injection on day 1 and **(C)** PTZ and CNO injection on day 2. Comparison of the percentage of different types of seizures in PV^Cre^/Gq-DREADD (DREADD) (*n* = 7) and non-DREADD WT control (*n* = 5) animals of the SScortex and the RTN thalamus group after **(B)** PTZ injection on day 1 and **(D)** PTZ and CNO injection on day 2.

The protocol and timeline for testing the impact of activating PV+ interneurons during PTZ-induced absence seizures is shown in Figures 3, 4. Experimental animals (implanted with either a cortical or thalamic cannula and scalp EEG electrodes) were injected with 20 mg/kg (i.p.) on day 1 to establish baseline seizure EEG profile; then the same cohort of animals were injected on day 2 with both PTZ (20 mg/kg) and CNO (5 mg/kg) focally delivered to ROI to activate FFI microcircuits within the CTC network during seizures.

#### PTZ Injection on Day 1 Induced Absence-Like Seizures and Other Types of Generalized Seizures in Both DREADD and Non-DREADD Mice

**FIGURE 5:**
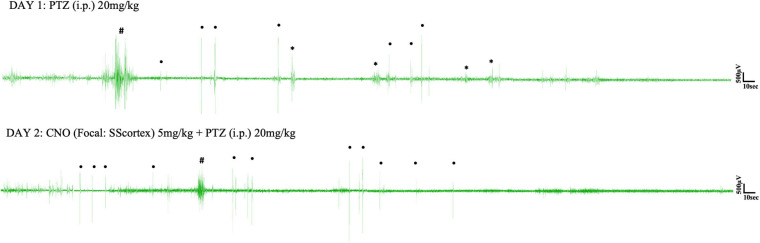
Representative EEG traces from a PV^Cre^/Gq-DREADD animal after i.p. PTZ injection on day 1 and focal (SScortex) CNO and i.p. PTZ injection on day 2. Asterisks (^∗^), hash signs (#), and dot signs (•) represent absence-like, tonic-clonic and other types of seizures, respectively. Each trace represents 10 min of EEG recording.

**FIGURE 6:**
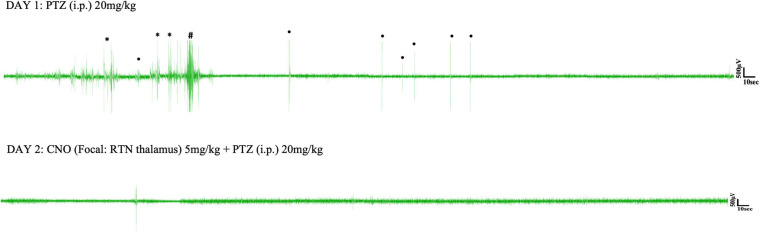
Representative EEG traces from a PV^Cre^/Gq-DREADD animal after i.p. PTZ injection on day 1 and focal (RTN thalamus) CNO and i.p. PTZ injection on day 2. Asterisks (^∗^), hash signs (#), and dot signs (•) represent absence-like, tonic-clonic and other types of seizures, respectively. Each trace represents 10 min of EEG recording.

Pentylenetetrazol (20 mg/kg) i.p. injection on day 1 induced epileptic seizures in all surgically implanted test animals. However, not all animals exhibited absence seizures. Tonic-clonic and other types of seizure were also seen in EEG (Figures 5, 6). In the SScortex group, 20 mg/kg PTZ induced absence seizures in 5 out of 7 PV^Cre^/Gq-DREADD and 3 out of 5 non-DREADD WT controls (Table 2). Six of the 7 DREADD mice and all 5 non-DREADD WT controls experienced tonic-clonic seizures. In the RTN thalamus cohort, absence seizures were observed in all DREADD mice (*n* = 7) and in 3 out of the 5 non-DREADD WT controls (Table 2). Tonic-clonic seizures occurred in only 3 out of 7 DREADD animals whereas all non-DREADD WT animals of this treatment group experienced tonic-clonic seizures.

**TABLE 2:** Number of animals exhibiting different seizure types on day 1 after PTZ administration.

Treatment group/Genotype (*n*)	No. of animals experiencing seizure types on day 1	
	Absence seizure	Tonic-clonic seizure
SScortex/DREADD (*n* = 7)	5	6
SScortex/non-DREADD (*n* = 5)	3	5
RTN thalamus/DREADD (*n* = 7)	7	3
RTN thalamus/non-DREADD (*n* = 5)	3	5

Analysis of EEG data after PTZ administration on day 1, showed that the first incident of an epileptic seizure (of any type) was very consistent (Figure 3B). In the SScortex group mean onset of seizures was 3.65 ± 0.63 min in PV^Cre^/Gq-DREADD mice (*n* = 7) and 3.59 ± 0.62 min in the non-DREADD WT control animals (*n* = 5). Similarly, in the RTN thalamus group seizures were induced 3.82 ± 0.74 min and 3.87 ± 1.03 min after PTZ injection in PV^Cre^/Gq-DREADD (*n* = 7) and non-DREADD WT control animals (*n* = 5), respectively. The time at which seizures terminated was also similar between the treatment groups (Figure 3C). The last seizure burst in SScortex group was 20.50 ± 7.87 min and 14.41 ± 6.03 min after PTZ injection in the PV^Cre^/Gq-DREADD (*n* = 7) and non-DREADD WT control animals (*n* = 5), respectively. Similarly, in the RTN thalamus group, last seizure burst was seen 13.07 ± 2.83 min and 14.27 ± 5.00 min after PTZ injection in DREADD (*n* = 7) and non-DREADD WT control animals (*n* = 5), respectively. So, in summary, there were no significant differences in the latency to the onset of first seizure or time of seizure termination between treatment groups and genotypes (Figures 3B,C) following a single injection of 20 mg/kg i.p. PTZ on day 1.

The relative time spent in PTZ-induced seizures during the 1 h EEG recording period on day 1 represented ≤2% of the total EEG test period (Figures 4A,B). The mean duration spent by DREADD SScortex group (*n* = 7) having absence-like seizures and tonic-clonic seizures was 17.85 ± 9.32 and 26.04 ± 11.15 s, respectively. Thus DREADD mice in the SScortex treatment group spent 36.13% of the overall seizure period having absence-like seizures and 52.70% experiencing tonic-clonic seizures (Figure 4C). Likewise, non-DREADD WT control animals spent 48.10% of total seizure period in absence seizures and 42.71% having tonic-clonic seizures (Figure 4C). The RTN thalamus group, DREADD animals (*n* = 7) displayed absence seizures for 31.11 ± 13.64 s and tonic-clonic seizures for 7.65 ± 2.96 s, representing 51.05 and 12.56%, respectively of the overall seizure period (Figure 4C). Non-DREADD WT animals in the thalamic group spent 30.92% of total seizure period displaying absence seizures and 49.23% having tonic-clonic seizures (Figure 4C). Overall, there were no significant differences in type and duration of seizures displayed by DREADD and non-DREADD cohorts on day 1 after PTZ administration (Figure 4C).

#### CNO Injection Into the SScortex or Thalamus on Day 2 Changed the Time Spent in Seizures by DREADD Mice but Not Non-DREADD Controls

Next we injected the same cohort of mice on day 2 with both PTZ and CNO to test the impact of activating feed-forward inhibitory PV+ interneurons during PTZ-induced seizures. Having established from day 1 baseline data that the seizure onset in PV^Cre^/Gq-DREADD and non-DREADD WT animals was approximately 5 min post-PTZ (20 mg/kg i.p.) injection, we first delivered CNO (5 mg/kg) focally into either the SScortex or the RTN thalamus 10 min prior PTZ (Figure 4C). The timing and dose of CNO were chosen based on data from our previous published report (Panthi and Leitch, 2019), which had established that 5 mg/kg CNO was the lowest effective dose that activated Gi-DREADD receptors and consistently generated absence-like seizures in PV^Cre^/Gi-DREADD mice ∼15 min post-CNO injection. Simultaneous video/EEG recordings were made according to the protocol outlined in schematic Figure 4C. The percentage of time spent in each of the different seizure types during 1 h of EEG recording on day 2 following CNO and PTZ administration, compared to day 1 data (PTZ only administration), is shown in Figures 4B,D.

On day 2, CNO activation of feedforward PV+ inhibitory interneurons in DREADD mice (both cortical and thalamic treatment groups), substantially changed the relative time spent having seizures. On day 1, PTZ-injected DREADD animals of the SScortex group (*n* = 7) spent 17.85 ± 9.32 and 26.04 ± 11.15 s in absence and tonic-clonic seizures, respectively. On day 2 after CNO and PTZ co-administration, animals spent less time in absence seizures (1.88 ± 1.87 s) and tonic-clonic seizures (2.88 ± 2.0 s) compared to day 1 (Figure 4D). Similarly, DREADD animals of the RTN thalamus group (*n* = 7) also spent less time in absence seizures on day 2 compared to that of day 1 (Figures 4B,D). During 1 h of EEG recording, these animals spent 31.11 ± 13.64 s in absence seizures on day 1 after PTZ administration. On day 2 after co-administration of CNO and PTZ, they spent only 5.49 ± 3.4 s in absence seizures (Figure 4D). Tonic-clonic seizures in this treatment group was also changed (day 1: 7.65 ± 2.96 s, day 2: 5.37 ± 3.53 s). Overall, DREADD animals spent significantly less time having either absence or tonic clonic seizures on day 2 compared to day 1 after activation of FFI. In the SScortex group, the duration spent in other types of seizures remained almost unchanged on both days, whereas in RTN thalamus group, the percentage time spent in other types of seizures was also substantially decreased on day 2 (Figures 4B,D).

In non-DREADD WT controls, CNO activation of feedforward PV+ inhibitory interneurons on day 2 did not reduce the overall time spent having mixed seizures; rather the percentage time spent having seizures was increased compared to day 1 (Figures 4C,D). However, the profile of individual seizure types varied. In the thalamus group, the percentage time animals spent in absence and tonic clonic seizures was increased compared to day 1; whereas, in the SScortex group tonic clonic seizures were increased compared to absence seizures. While it is unclear why the seizure profile changed with respect to this group, nevertheless, the overall impact of CNO and PTZ co-administration on day 2 had no antiepileptic effects in non-DREADD WT control mice (Figures 4B,D).

##### Activating PV+ interneurons with CNO either prevented or delayed the onset of PTZ-induced seizures in all DREADD mice tested but had no impact on non-DREADD controls

**FIGURE 7:**
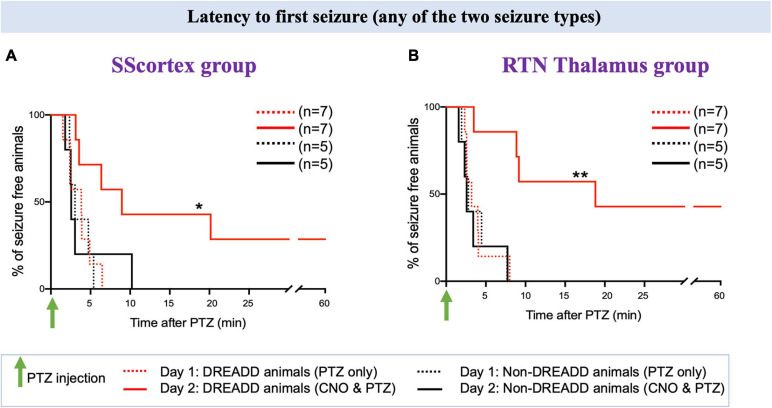
Comparison of the latency to first seizure (any of the two seizure type) in PV^Cre^/Gq-DREADD and non-DREADD WT controls of the **(A)** SScortex group and the **(B)** RTN thalamus group between day 1 (PTZ only treated) and day 2 (CNO and PTZ treated). Comparisons between the treatment groups were made using log-rank test. **(A)** *p = 0.0477; **(B)** **p = 0.0019.

Activating PV+ interneurons in the SScortex with CNO on day 2 prevented seizures in 2 out of 7 DREADD animals in this group i.e., 29% did not experience any type of seizure during 1 h of EEG recording (Figure 7A, solid red line). Likewise, in the RTN thalamus group, CNO activation of PV+ interneurons suppressed seizures in 3 out of 7 (42%) DREADD mice (Figure 7B solid red line). In the remaining PV^Cre^/Gq-DREADD animals of both groups, the latency to first seizure was delayed. In the SScortex group, mean latency to first seizure was 4.32 ± 0.55 min (*n* = 5) on day 1 (Figure 7A, dashed red line), which increased to 8.43 ± 31 min (*n* = 5) on day 2 (Figure 7A, solid red line). Likewise, in the RTN thalamus group on day 1, mean onset of seizure was 3.24 ± 0.34 min (*n* = 4; Figure 7B, dashed red line), which increased to 10.08 ± 3.19 min (*n* = 4) on day 2 (Figure 7B, solid red line). In contrast, activation of PV+ interneurons did not delay the latency to first seizure in non-DREADD WT control animals [SScortex group, day 1: 3.59 ± 0.62 min (*n* = 5), day 2: 4.03 ± 1.56 min (*n* = 5); RTN thalamus, day 1; 3.87 ± 1.03 min (*n* = 5), day 2: 3.51 ± 1.09 min (*n* = 5); Figures 7A,B black lines]. A log-rank test was performed to statistically compare the latency to the first seizure between treatment groups (Figures 7A,B). This test indicated that the latency to first seizure (of any type) was significantly delayed in PV^Cre^/Gq-DREADD animals of both SScortex and RTN thalamus groups on day 2 compared to that of day 1 but not in the non-DREADD WT controls (Figures 7A,B).

##### Latency to first absence and tonic-clonic is delayed in DREADD mice

**FIGURE 8:**
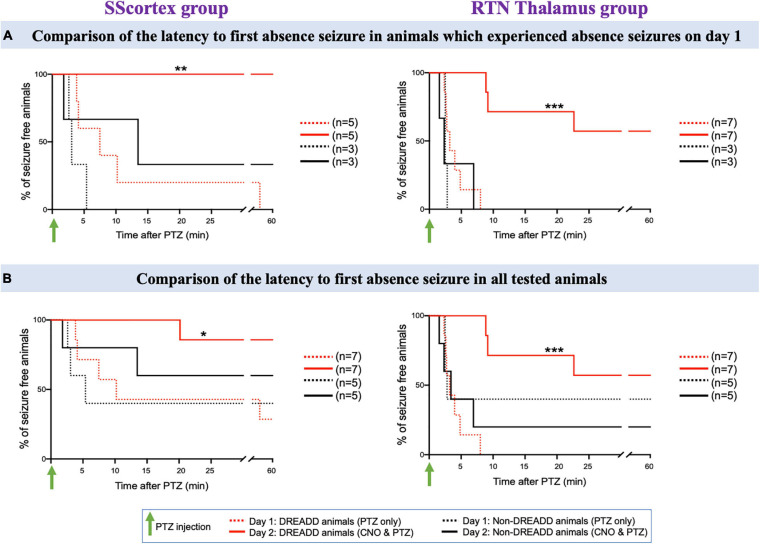
**(A)** Comparison of the latency to first absence seizure in animals which experienced absence seizures on day 1. **(B)** Comparison of the latency to first absence seizure in all tested PV^Cre^/Gq-DREADD and non-DREADD WT controls of the SScortex group and the RTN thalamus group between day 1 (PTZ only treated) and day 2 (CNO and PTZ treated). Comparisons between the treatment groups were made using log-rank test. **(A)** **p = 0.0024, ***p = 0.0002; **(B)** *p = 0.0268, ***p = 0.0001.

**FIGURE 9:**
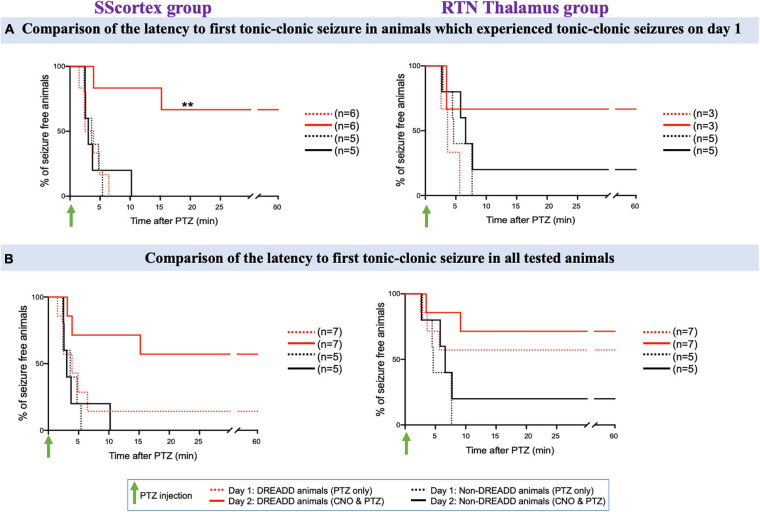
**(A)** Comparison of the latency to first tonic-clonic seizure in animals, which experienced tonic-clonic seizures on day 1. **(B)** Comparison of the latency to first tonic-clonic seizure in all tested PV^Cre^/Gq-DREADD and non-DREADD WT controls of the SScortex group and the RTN thalamus group between day 1 (PTZ only treated) and day 2 (CNO and PTZ treated). Comparisons between the treatment groups were made using log-rank test. **p = 0.0067.

**FIGURE 10:**
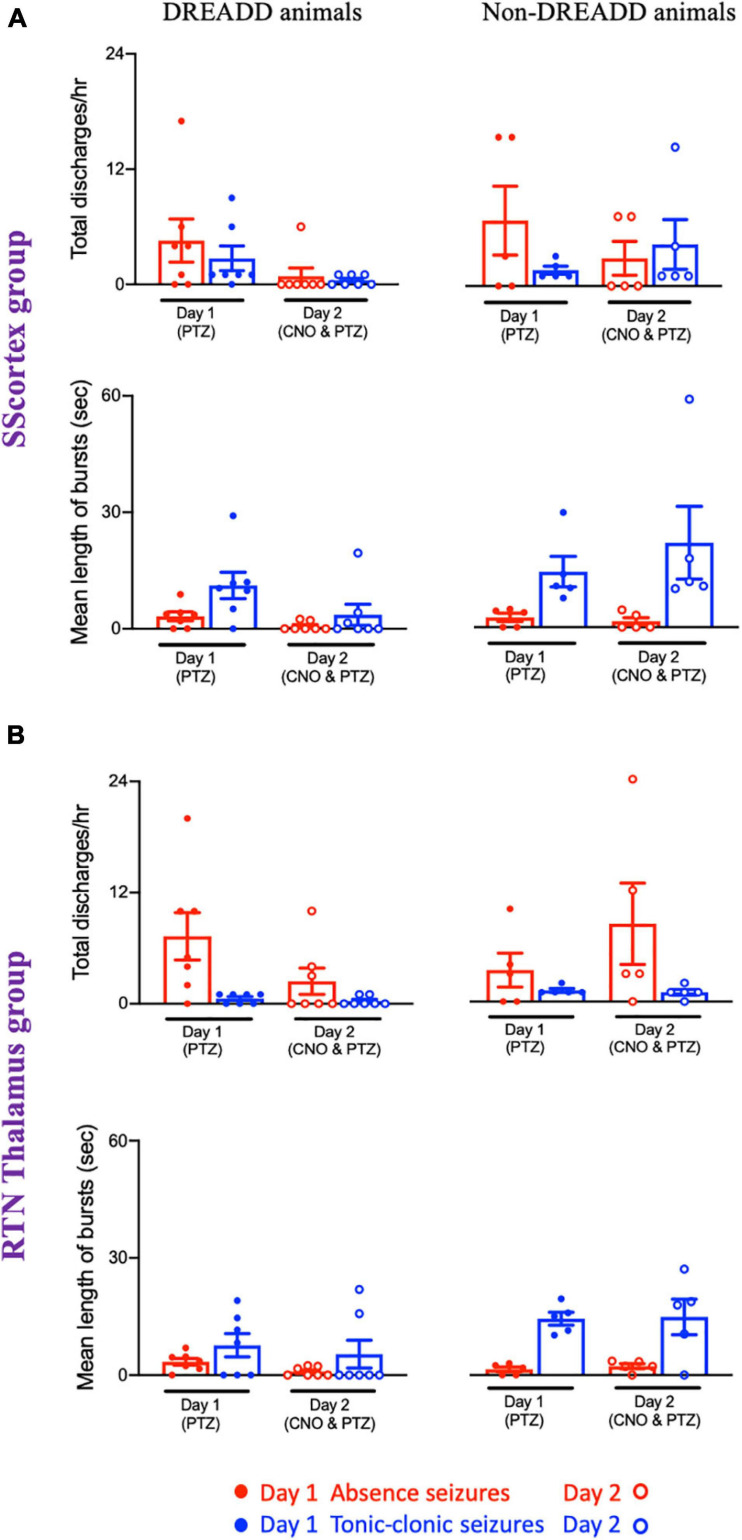
Comparison of mean number of epileptic bursts (discharges/h) and mean length of bursts between PV^Cre^/Gq-DREADD (DREADD) (*n* = 7) and non-DREADD WT (*n* = 5) animals of **(A)** the SScortex and **(B)** the RTN thalamus group after PTZ treatment on day 1 and CNO and PTZ treatment on day 2. All values represent mean ± SEM. Comparisons were performed using Wilcoxon matched-pairs signed-rank test.

We further analyzed the EEG data to determine the latency to onset of each type of seizure. Latency to first absence or tonic-clonic seizure in PTZ injected PV^Cre^/Gq-DREADD animals pre-treated with CNO (in either of the brain regions of CTC network) on day 2 was delayed compared to that of day 1 (Figures 8–10).

###### Absence seizures

First, we compared the latency to first absence seizure on day 2 in those mice that had EEG evidence of absence seizures on day 1 (Figure 8A); and then in all tested animals (Figure 8B). In the SScortex group on day 1, PTZ induced absence seizures in 5 PV^Cre^/Gq-DREADD animals. All 5 animals (100%) were completely absence seizure free when CNO and PTZ were co-administered on day 2 (Figure 8A, solid red line). Of the remaining 2 mice in this cohort that didn’t have absence seizures on day 1, one had absence seizures on day 2 but delayed (i.e., 20.15 min post-PTZ and CNO co-administration compared to mean latency to first absence seizures on day 1 i.e., 13.52 ± 6.04 min) and the other had no absence seizures either on day 1 or day 2 (Figure 8B). In DREADD animals of RTN thalamus group, 4 out of 7 (57%) animals did not experience absence seizure on day 2 (Figure 8A solid red line); whereas all seven had displayed SWDs on EEG on day 1 (Figure 8A dashed red line). In the remaining three animals, mean latency to first absence burst on day 2 was also delayed compared to that of day 1 i.e., day 1: 3.71 ± 0.73 min; day 2: 13.55 ± 4.54 min. Overall, there was a significant reduction in absence seizures and also latency to first absence seizure in DREADD animals treated with CNO to activate FFI in both the SScortex and thalamus (Figures 8A,B asterisks). In contrast no significant difference in absence seizures was evident in non-DREADD controls treated with CNO.

###### Tonic-clonic seizures

The latency to first tonic-clonic seizure in animals, which experienced this type of seizure on day 1, is shown in Figure 9A and the latency to first tonic-clonic seizure in all tested animals is shown in Figure 9B. PTZ treatment induced tonic-clonic seizures in 6 DREADD animals of the SScortex group on day 1; activation of feed-forward inhibitory PV+ interneurons on day 2 prevented tonic-clonic seizures in 4 (67%) of these mice (Figure 9A, solid red line). The remaining animals showed a delayed onset of tonic clonic-seizures compared to that of day 1 i.e., day 1: 3.69 ± 1.19 min; day 2: 9.56 ± 5.64 min (Figure 9A). In contrast CNO activation of FFI in the SScortex of non-DREADD controls had no impact on tonic-clonic seizures with all mice in this cohort experiencing tonic-clonic seizures within ∼10 min on both days mice (Figures 9A,B; black line). In the RTN thalamus group, PTZ treatment produced tonic-clonic seizures in 3 out of 7 DREADD animals on day 1. Upon co-administration of CNO and PTZ, on day 2, 67% animals did not experience any tonic-clonic seizures (Figure 9A). In the non-DREADD controls treated with CNO (*n* = 5), only one animal was free of tonic-clonic seizures on day 2.

##### Activating PV+ interneurons reduced total number of discharges and mean length of epileptic bursts

Analysis of the EEG data showed that total number of discharges (absence and tonic-clonic bursts) in PV^Cre^/Gq-DREADD mice of both SScortex group and the RTN thalamus group was reduced on day 2 compared to that of day 1 (Figure 10). PV^Cre^/Gq-DREADD animals of SScortex group (*n* = 7) treated with PTZ exhibited 4.57 ± 2.24 absence discharges, which was reduced to 0.85 ± 0.85 discharges on day 2 (Figure 10A). Similarly, these animals experienced 2.71 ± 1.28 tonic-clonic discharges on day 1, which were reduced to 0.42 ± 0.205 tonic-clonic discharges on day 2 (Figure 10A). Similar effects were seen in RTN thalamus group. PV^Cre^/Gq-DREADD animals of this group (*n* = 7) treated with PTZ displayed 7.42 ± 2.58 absence-like discharges on day 1, which was reduced to 2.42 ± 1.41 discharges on day 2 (Figure 10B). Those animals also displayed 0.57 ± 0.20 tonic-clonic discharges on day 1, which were reduced to 0.28 ± 0.18 tonic-clonic discharges on day 2 (Figure 10B). In contrast, no reduction in total number of discharges was evident in non-DREADD WT control animals (Figures 10A,B).

Analysis of the EEG data also showed that, there was a reduction in mean duration of each epileptic bursts in PV^Cre^/Gq-DREADD animals on day 2 compared to that of day 1 (Figures 10A,B). In the SScortex group, the mean length of absence and tonic-clonic seizures was 3.21 ± 1.15 and 11.18 ± 3.40 s, respectively, which decreased to 0.66 ± 0.43 and 3.60 ± 2.71 s on day 2 (Figure 10A). Similar reductions were also seen in the PV^Cre^/Gq-DREADD animals of RTN thalamus group. PV^Cre^/Gq-DREADD animals of the RTN thalamus group on day 1 displayed absence and tonic-clonic seizures spanning 3.42 ± 0.86 and 7.65 ± 2.96 s, which was reduced to 0.90 ± 0.43 and 5.37 ± 3.53 s on day 2 (Figure 10B). Overall, PV^Cre^/Gq-DREADD animals of both treatment groups experienced a reduction in the number of epileptic discharges and mean length of such discharges on day 2, whereas non-DREADD WT control animals of both treatment groups did not show reductions in either number or duration of epileptic bursts (Figures 10A,B).

## Discussion

In this study, we used DREADD technology to investigate the impact of activating FFI within the CTC network during chemically (PTZ) induced seizures. We show that excitation of feed-forward inhibitory PV+ interneurons within the SScortex and RTN (via CNO activation of Gq-DREADD receptors) during PTZ-induced seizures has antiepileptic effects. Analysis of the individual seizure types revealed that activation of FFI was effective against PTZ-induced absence seizures and also other forms of generalized seizures evoked by PTZ i.p. administration. Such activation either prevented or delayed the latency to first seizure, decreased total number of discharges and their mean duration, and reduced the overall time spent in seizures. In contrast, focal injection of CNO into either the SScortex or RTN thalamus of non-DREADD WT control animals, had no effect on PTZ-induced seizures. These data demonstrate a potential for using PV+ interneurons as a therapeutic target to control absence seizures in some cases of human absence epilepsy.

### Excitatory Gq-DREADD Receptors Are Highly Expressed in Feed-Forward Inhibitory PV+ Interneurons in PVCre/Gq-DREADD Mice

Confocal immunofluorescence microscopy confirmed the expression of excitatory (Gq) DREADD receptors in PV+ interneurons in PV^Cre^/Gq-DREADD animals. DREADD HA-tag was highly expressed in all cells known to contain PV. The levels of co-localization were greater than 90% in PV+ cells in the SScortex, RTN thalamus and the Purkinje cells of the cerebellum in PV^Cre^/Gq-DREADD mice. In contrast, none of the non-DREADD WT control animals showed any HA-tag label in PV+ cells. The labeling of PV+ cells in this study was consistent with that of other published studies (del Río and DeFelipe, 1994; Tamamaki et al., 2003; Dávid et al., 2007; Fishell, 2007; Xu et al., 2010). The efficacy and specificity of this double transgenic approach for expressing DREADD receptors in feed-forward inhibitory PV+ interneurons was higher than that reported for viral-mediated DREADD expression in PV-Cre animals. Studies have shown that the infection efficiency and specificity of labeling vary between 70 and 95% in viral-mediated delivery of DREADD constructs into brain regions (Zou et al., 2016; Xia et al., 2017; Hijazi et al., 2019; Bicks et al., 2020). In our current study, the percentage of co-labeling of HA-tagged Gq-DREADD receptors and PV was always above 90% in all brain regions analyzed. This is similar to the results in our previous published report (Panthi and Leitch, 2019) for inhibitory Gi-DREADD receptor expression in PV+ cells.

### Activation of Feed-Forward Inhibitory PV+ Interneurons in the CTC Network Suppressed PTZ-Induced Absence Seizures

Pentylenetetrazol is one of the chemicals that is widely used to induce generalized absence seizures in animals. Other chemicals such as γ-hydroxybutyrate (GHB), bicuculline, picrotoxin, penicillin are also routinely used to induce absence seizures (Snead, 1992; Cortez et al., 2016; Kostopoulos, 2017), but PTZ has been preferentially used for testing new drugs against absence seizures for more than 70 years (see reviews by Krall et al., 1978; Löscher, 2011). Moreover, use of low dose of PTZ is an established method to induce generalized seizures within the thalamocortical circuit (Snead, 1992; Snead et al., 2000; Cortez et al., 2016).

In the pilot study, a single 20 mg/kg (i.p.) dose of PTZ was needed to induce absence seizures in PV^Cre^/Gq-DREADD and non-DREADD mice. However, a dose of 20 mg/kg also induced tonic-clonic seizures. Higher doses of PTZ (30 mg/kg and above) induced severe tonic-clonic seizures. Other studies have also reported that the severity and behavioral features of PTZ-induced seizures vary with the concentration/dose of PTZ, also genotype and age of mouse (Van Erum et al., 2020). Similar dose/response variability have also been reported in rats (Klioueva et al., 2001; Lüttjohann et al., 2009). In our study seizures were categorized on the basis of their EEG waveform profile and behavioral features during simultaneous EEG/video recordings; notably SWD and behavioral arrest were classified as absence seizures. Administration of 20 mg/kg PTZ has been reported to induce SWDs with behavioral arrest in Tau58/4 transgenic mice (Van Erum et al., 2019). Likewise, i.p. administration of 25 mg/kg PTZ into leptin-deficient mice and their wild-type counterparts induced absence seizures, myoclonic seizures, generalized clonic and clonic–tonic seizures but the proportion of absence seizures were higher (Erbayat-Altay et al., 2008). Importantly, in our current study, the proportion of absence and tonic-clonic seizures after PTZ treatment (i.p., 20 mg/kg, on day 1) in PV^Cre^/Gq-DREADD and non-DREADD WT control animals was not significantly different. Thus, it can be concluded that injection of low dose PTZ (20 mg/kg) in our study was optimum for inducing absence seizures (albeit mixed with some other seizure types) in both PV^Cre^/Gq-DREADD and non-DREADD mice to test the impact of FFI on absence seizure generation and severity.

The latency to first seizure after PTZ treatment (on day 1) was within 5 min in PV^Cre^/Gq-DREADD and non-DREADD mice, which is consistent with other published studies in mice (Keskil et al., 2001; Medina et al., 2001; Ilhan et al., 2006; Erbayat-Altay et al., 2008; Nassiri-Asl et al., 2009; Koutroumanidou et al., 2013; Faghihi and Mohammadi, 2017; de Freitas et al., 2018). These studies have shown that irrespective of dose of PTZ (20–80 mg/kg, i.p.), the latency of seizure onset is within 5 min. However, some other studies have reported that increasing the concentration of PTZ from 40 to 70 mg/kg, substantially decreases the time of onset of first observed seizure from ∼5 min to ∼1 min (Medina et al., 2001; Schwaller et al., 2004). Likewise, Girard et al. (2019) found that latency to seizure was decreased in mice injected with 60 mg/kg compared to animals injected with PTZ doses of 30–50 mg/kg. Van Erum et al. (2020) recently reported genotype and age-related differences in susceptibility and onset of PTZ-induced seizures in mice. However, in the current study, regardless of genotype, first and last incident of seizure after 20 mg/kg PTZ administration on day 1, was very consistent in all animals of both treatment groups (PV^Cre^/Gq-DREADD and non-DREADD mice surgically implanted with either cortical or thalamic cannulae).

On day 2, PTZ (20 mg/kg, i.p.) was tested after pre-treatment with CNO (5 mg/kg, into either the SScortex or the RTN thalamus). The timing of CNO treatment was based on the evidence from previous published reports where activating PV+ interneurons during pre-ictal stage provided anti-epileptic effects but activating them during the interictal phase induced epileptic events (Yekhlef et al., 2015; Assaf and Schiller, 2016). The exact timing and dose of CNO is previously explained in section “CNO Injection Into the SScortex or Thalamus on Day 2 Changed the Time Spent in Seizures by DREADD Mice but Not Non-DREADD Controls.” In this study, focal activation of PV+ interneurons in either cortical or thalamic microcircuits on day 2 either prevented or significantly delayed the latency to first absence seizure (and another seizure type) compared to day 1 seizure onset in PTZ treated PV^Cre^/Gq-DREADD animals. Furthermore, there was a reduction in the number of SWDs and mean duration of each burst of discharges and the total time spent having seizures, on day 2. However, co-administration of CNO and PTZ in non-DREADD WT control animals of both treatment groups (SScortex and RTN thalamus) on day 2 did not decrease total time spent in seizures compared to day 1. Other parameters such as total discharges and mean length of bursts in non-DREADD WT control animals also did not remarkably change upon co-administration of CNO and PTZ on day 2, compared to day 1. Thus is can be concluded that unilateral activation of FFI, within the CTC network (by CNO activation of Gq-DREADD receptors in PV+ interneurons) during PTZ-induced seizures suppresses or reduces the severity of absence seizures and other types of generalized seizure. These findings are in agreement with those from a study by Clemente-Perez et al. (2017) who found that optogenetic activation of PV+ interneurons, but not somatostatin expressing inhibitory neurons, in the RTN thalamus suppressed PTZ-induced (35–60 mg/kg) spike-and-wave seizures seen in EEG and spiking seizure episodes observed in thalamic local field potential (LFP) recordings.

Activation of PV+ interneurons in other brain regions has also been shown to afford protection against different types of chemically induced seizures. DREADD-mediated activation of hippocampal PV+ interneurons suppressed 4-AP induced *in vivo* convulsive behavior and also *in vitro* epileptiform discharges in PV-Cre mice (Cǎlin et al., 2018), Similarly, activation of hippocampal PV+ interneurons provided antiepileptic effects against KA-induced temporal lobe seizures in mice (Krook-Magnuson et al., 2013; Wang et al., 2018). In another study, Ledri et al. (2014) found that simultaneous optogenetic activation of several subpopulation of interneurons in the hippocampus [PV, SOM, Cholecystokinin (CCK), Neuropeptide Y (NPY)-expressing] was more effective in inhibiting 4-AP induced epileptiform activity in brain slices compared to activation of individual classes of interneurons. However, it should be noted that, in some studies, activation of PV+ interneurons did not stop or shorten chemically induced seizures as expected. For example, activation of PV+ interneurons increased epileptic discharges induced by 4-AP (Yekhlef et al., 2015). In another study by Khoshkhoo et al. (2017) it was found that silencing PV+ interneurons in the primary motor cortex was effective in reducing the duration of optogenetically induced electrographic seizures (Khoshkhoo et al., 2017). Nevertheless, global activation of PV+ interneurons (using DREADD technology) has been shown to reduce the severity of PTZ (50 mg/kg) induced tonic-clonic and myoclonic seizures in transgenic PV-Cre × LSL-hM3Dq mice (Johnson et al., 2018). Furthermore, the protective role played by inhibitory feedforward PV+ interneurons in preventing runaway excitation and seizures is confirmed by studies in PV knockout (PV^–/–^) mice. These PV^–/–^ mice had more severe PTZ-induced seizures compared to their wild-type (PV^+/+^) genotype controls (Schwaller et al., 2004). Collectively, these findings indicate that PV+ interneurons might serve as a therapeutic target to control seizures.

Functional recordings from PV+ interneurons of stargazer animals would be highly beneficial to determine the degree to which the impaired AMPA receptor might have been involved in the alteration of activity of PV+ interneurons, as previous studies conducted in epileptic stargazers showed abnormal expression of AMPA receptors particularly at input synapses of CTC PV+ neurons (Barad et al., 2012; Maheshwari et al., 2013; Adotevi and Leitch, 2016, 2017, 2019). However, there is a technical limitation. The distance between the stargazin locus to parvalbumin locus in chromosome 15 is very close (separated by only 0.01 cM) and if stargazers are bred with parvalbumin promoter-driven animals, double crossover is very unlikely and identification of these cells during physiological study would be technically challenging (Maheshwari et al., 2013). Use of viral vector mediated delivery of the DREADD receptors into the CTC regions in other genetic rodent models of absence epilepsy (e.g., GAERS or WAG/Rij rats) is however possible and potentially could be helpful to interrogate the role of PV+ interneurons on absence seizures in future studies.

### PV+ Interneurons–A Potential Target for Anti-Epileptic Therapy?

In our previous published report (Panthi and Leitch, 2019), we demonstrated that selective silencing of feed-forward inhibitory PV+ interneurons in the CTC network via regional injection of CNO into either the SScortex or the RTN thalamus of PV^Cre^/Gi-DREADD mice, generated absence-like SWDs. In the current study, we further demonstrate that selectively activating these interneurons, during PTZ-induced seizures, prevents or suppresses the severity of absence seizures. Furthermore, PTZ-induced tonic-clonic seizures are also reduced in severity by activation of feed-forward inhibitory PV+ interneurons within the CTC network. PV+ interneurons account for ∼40% of the GABAergic population (Xu et al., 2010). Most PV+ neurons in the cortex are basket and chandelier cells; they are found throughout cortical layers 2–6. PV+ interneurons, typically have fast-spiking, low input resistance, and high-amplitude rapid after-hyperpolarization characteristics (Kawaguchi et al., 1987; Kawaguchi and Kubota, 1997), which enables them to fire a rapid train of action potentials unlike any other neuron in the cortex. They are therefore likely to have a profound impact on the spiking output of their targets (for review see Ferguson and Gao, 2018).

In the SScortex, PV+ interneurons synapse on to soma/proximal dendrites/axon initial segment of excitatory pyramidal cells (Inan and Anderson, 2014; Tremblay et al., 2016). PV+ interneurons are densely connected to pyramidal cells across cortical layers and areas influencing their excitability (Packer and Yuste, 2011; Hu et al., 2014). A single PV interneuron contacts nearly every local pyramidal neuron (Ferguson and Gao, 2018). Packer and Yuste (2011) estimated that a typical PV+ interneuron in the cortex makes contact with hundreds to thousands of post-synaptic targets (both pyramidal cells and other PV+ interneurons) and each excitatory pyramidal cell is contacted by ∼50–200 inhibitory PV+ interneurons. Thus activating them may generate widespread post-synaptic inhibitory currents in pyramidal neurons, thereby preventing cortical hyper-excitation and attenuating PTZ-induced absence seizures.

On the other hand, PV+ interneurons in the RTN project onto excitatory thalamocortical relay neurons of VP thalamus and provide powerful feedforward inhibition (Fogerson and Huguenard, 2016); thus activating them may have prevented the firing of excitatory relay neurons of VP thalamus, thereby disrupting generalized absence-SWDs induced by systemic PTZ injection.

It is important to note that a number of different molecular mechanisms are capable of switching CTC network from normal oscillations to pathological absence-SWD oscillations. There is a long-standing debate over the site of initiation of absence-SWDs within the CTC network. However, the current general consensus is that rhythmic epileptic discharges are initiated in the cortex (for reviews see Meeren et al., 2005; Crunelli et al., 2020). Meeren et al. (2002) were the first researchers to clearly demonstrate the initiation of absence seizures in the perioral region of primary SScortex of (WAG/Rij) rats. They showed that the SScortex always leads all other cortical and thalamic sites during the first ∼500 ms of absence seizures; but after cortical initiation, the activity of either thalamic or cortical neurons may precede the other during subsequent paroxysmal cycles. Later studies on GAERS rats confirmed the primary SScortex (Studer et al., 2018) and specifically layer 5/6 excitatory pyramidal neurons (Polack et al., 2007), as the originators of the initial paroxysmal activity. It is now accepted that a cortical initiation network (CIN) contributes to the pre-ictal changes of absence seizure.

Notwithstanding, once initiated both the cortex and thalamus are intimately involved in the generation and maintenance of SWDs. McCafferty et al. (2018) have demonstrated that both cortex and thalamus consistently receive a robust burst of action potentials from the other region (in correlation with EEG spikes) at each and every SWD cycle. The critical role played by the thalamus is also indicated by the fact that SWD and absence seizures can be induced experimental by activation of different thalamic regions in non-epileptic animals (Avoli, 2012; Lüttjohann and van Luijtelaar, 2015; Sorokin et al., 2017). Numerous experimental studies have demonstrated that changes in neuronal excitability limited to a restricted cortical or thalamic region can lead to generalized SWD-like activity throughout the CTC network (Tan et al., 2007; Rossignol et al., 2013; Crunelli et al., 2020). In this context it is interesting to note that in our previous study (Panthi and Leitch, 2019), acute unilateral silencing of PV+ interneurons in either cortical or thalamic regions was sufficient to illicit SWDs; likewise the current study shows that activation of these same PV+ interneurons (either cortical or thalamic) during chemically induced seizures, profoundly decreases SWDs expression and the generation and severity of absence seizure.

## Conclusion

Collectively, our data indicate that DREADD-mediated activation of feed-forward inhibitory PV+ interneurons, in either somatosensory cortical or thalamic microcircuits of the CTC network, yields antiepileptic effects against chemically induced absence seizures. Thus, these interneurons might serve as targets for anti-seizure therapy for some forms of absence epilepsy. The findings from this study could be highly significant in developing new targeted approaches for the treatment of childhood absence epilepsy in patients from different genetic backgrounds.

## Data Availability Statement

The raw data supporting the conclusions of this article will be made available by the authors, without undue reservation.

## Ethics Statement

All experiments were performed in accordance with the University of Otago Animal Ethics Committee under the AEC no. D94/16 and AUP-19-98.

## Author Contributions

BL was responsible for conception, hypothesis development and design of the research, also secured the funding, and wrote the manuscript. SP conducted the experiments and data analyses. Both authors conducted the interpretation of the results, contributed to draft versions of the manuscript, and approved the final version.

## Conflict of Interest

The authors declare that the research was conducted in the absence of any commercial or financial relationships that could be construed as a potential conflict of interest.

## Footnotes

1 https://www.jax.org/strain/026220

2 https://www.jax.org/strain/008069

## Supplementary Material

Supplementary Figure 1 **(A)** Schematic showing breeding paradigm to generate PV-Cre × hM3Dq-flox offspring and WT (non-DREADD) control mice by crossing a female homozygous PV-Cre mouse with heterozygous hM3Dq-floxed mice. **(B)** Representative gel electrophoresis blot showing the homozygous (Homo) PV-Cre knockin (350 and 300 bp) and the hM3Dq-flox [heterozygous (Het) 300 and 204 bp and wild-type (WT) 300 bp] for three mice to verify PV-Cre knockin and hM4Di-flox. For the confirmation of PV-Cre knockin and hM3Dq-flox in the same mouse, genotyping was performed separately.

Supplementary Figure 2 Representative coronal slices of mouse brain showing histological localization of methylene blue dye injected via a cannula located in either **(A)** SScortex and **(B)** thalamus, verifying CNO site of diffusion within those regions. Coronal slice drawings were adapted from Mouse Brain Atlas.
