# Supplementary figures and images for: Chemogenetic Activation of Feed-Forward Inhibitory Parvalbumin-Expressing Interneurons in the Cortico-Thalamocortical Network During Absence Seizures
Source: Front Cell Neurosci. 2021 May 28;15:688905. doi: 10.3389/fncel.2021.688905 (PMC8193234; doi:10.3389/fncel.2021.688905)

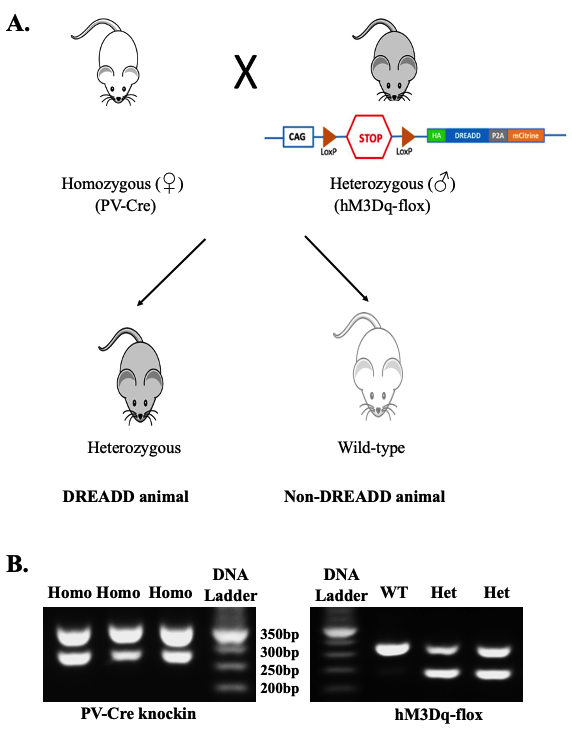

Supplement: Supplementary Figure 1 — (A) Schematic showing breeding paradigm to generate PV-Cre × hM3Dq-flox offspring and WT (non-DREADD) control mice by crossing a female homozygous PV-Cre mouse with heterozygous hM3Dq-floxed mice. (B) Representative gel electrophoresis blot showing the homozygous (Homo) PV-Cre knockin (350 and 300 bp) and the hM3Dq-flox [heterozygous (Het) 300 and 204 bp and wild-type (WT) 300 bp] for three mice to verify PV-Cre knockin and hM4Di-flox. For the confirmation of PV-Cre knockin and hM3Dq-flox in the same mouse, genotyping was performed separately. [file Image_1.TIFF]

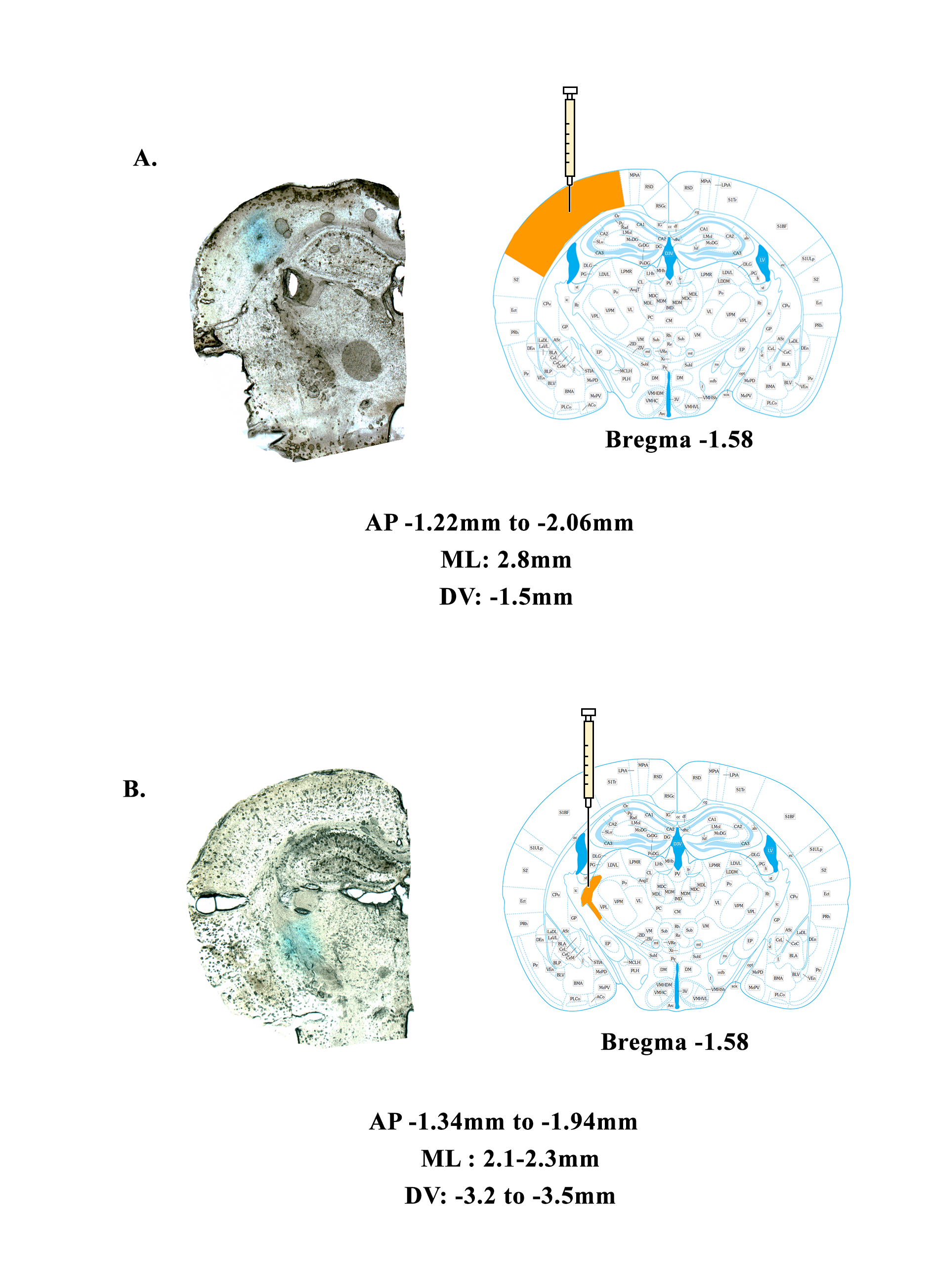

Supplement: Supplementary Figure 2 — Representative coronal slices of mouse brain showing histological localization of methylene blue dye injected via a cannula located in either (A) SScortex and (B) thalamus, verifying CNO site of diffusion within those regions. Coronal slice drawings were adapted from Mouse Brain Atlas. [file Image_2.TIFF]
